# Development of an Anatomical Silicone Model for Simulation-based Medical Training of Obstetric Anal Sphincter Injury Repair in Bangladesh

**DOI:** 10.7759/cureus.3991

**Published:** 2019-01-31

**Authors:** Christine Goudie, Atamjit Gill, Jessica Shanahan, Andrew Furey, Adam Dubrowski

**Affiliations:** 1 Medical Education and Simulation, Memorial University of Newfoundland, St. John's, CAN; 2 Obstetrics and Gynecology, Memorial University of Newfoundland, St. John's, CAN; 3 Orthopaedics, Memorial University of Newfoundland, St. John's, CAN; 4 Emergency Medicine, Memorial University of Newfoundland, St. John's, CAN

**Keywords:** simulation, 3d printing, obstetrics and gynaecology, anal sphincter, oasis, postpartum, perineal repair, developing countries, international health, simulation based medical education

## Abstract

Advancing global healthcare in developing countries has traditionally been an area of interest for many North American medical organizations, as they strive to improve patient outcomes by helping to control disease and death-related illnesses. Women’s healthcare in developing countries, in particular, presents a unique set of complexities, revealing high maternal mortality statistics surrounding pregnancy, labor, and childbirth, which is often tied to home births without medically trained attendants. In September 2018, Team Broken Earth, a Canadian-based outreach initiative, hosted a three-day women’s healthcare course in Dhaka, Bangladesh, which included simulation-based training stations, for the purpose of advancing clinical skills and education in regards to local labor and delivery. The training stations included the prevention of shoulder dystocia, helping babies breathe, the application of uterine compression sutures, and the repair of obstetric anal sphincter injuries (OASIS). The OASIS management station provided an opportunity to practice anal sphincter repair on anatomically accurate silicone models, which was a focus of the training course due to the high frequency of such injuries in rural Bangladesh. Evaluation surveys were supplied to workshop participants to capture their feedback about the use of the OASIS models and their efficacy as a training tool in Bangladesh.

Overall, the models were considered superior as compared to pre-existing training methods, which traditionally involve textbook education and hands-on learning in emergency birthing scenarios by non-medically trained attendants. Two minor iterative improvements were suggested during the Team Broken Earth workshops in Dhaka, Bangladesh, with regards to improving the models for future use: (a) the ethnicity coloring of the models should be more inclusive, especially when delivering training in international countries, and (b) future silicone models should include the addition of mesh across the bottom layer to ensure participants fingers did not rupture the enclosed vaginal canal while suturing. The purpose of this technical report is to determine the efficacy of a silicone OASIS model, developed for practicing high-risk laceration repair that can occur during childbirth, which presents in higher frequency in developing countries, such as Bangladesh, due to the number of rural at-home deliveries.

The original study in this series involved the investigation of silicone perineal repair models focusing on first- and second-degree lacerations, which were used at the Remote and Rural Conference in St. John’s, Newfoundland, in April 2018. The facilitators distributed the first iteration of the models to conference participants and collected participant feedback, which concluded that several improvements were required to enhance the models for medical training purposes. With the iterative revisions complete, the model is now under further validation testing to determine its efficacy within simulation-based medical education (SBME) and clinical skill maintenance. This technical report is the second in the series and includes the most recent third and fourth-degree silicone models as well as all suggested improvements from previous clinical feedback.

## Introduction

Advancing global healthcare in developing countries has traditionally been an area of interest for many North American medical organizations, as they strive to improve patient outcomes by helping to control disease and death- related illnesses [1]. In recent years, North American medical schools have also seen a surge in global health training for students and residents as well as an overarching interest to implement long-term sustainable healthcare programs as opposed to one-time medical missions [2]. Women’s healthcare in low resource environments, in particular, has been a major focus of many institutions, identifying a need to advance education and clinical hands-on training [3].

Organizations such as the Canadian-based Team Broken Earth, founded by Orthopedic Surgeon Dr. Andrew Furey, has committed to the advancement of such sustainable international relief efforts and women’s health education courses in such locations as Bangladesh, Guatemala, Nicaragua, Nepal, and Haiti [4]. Team Broken Earth comprises 12 teams across Canada, offering medical relief through emergency response, educational programs to advance international medical training, as well as ongoing advocacy support for each of its sites [5].

In September 2018, Team Broken Earth visited Dhaka, Bangladesh, to host the International Training Course on High-Risk Labour and Delivery Management, designed to provide hands-on simulation-based training for local clinicians in Dhaka. Women’s healthcare in Bangladesh has presented a unique set of complexities, presenting extreme birth injuries and elevated mortality statistics surrounding pregnancy and childbirth, linked to a high number of home births without medically trained attendants [6]. The three-day course included eight simulation-based training stations, which delegates rotated through for the purpose of improving various aspects of labor and delivery. The training stations included topics such as the prevention of shoulder dystocia, helping babies breathe, the management of obstetric anal sphincter injuries (OASIS), the utilization of three-dimensional (3D) printing for the purpose of producing simulation-based learning tools and task trainers, and the application of postpartum uterus compression sutures. The OASIS management station was the main focus of the training course as a method to combat maternal mortality rates in rural Bangladesh, which is linked to 70% of women giving birth at home with the assistance of non-medically trained attendees [7].

Twenty-five silicone OASIS perineal repair models were provided in-kind to Team Broken Earth by MUN Med 3D, a biomedical design and 3D printing laboratory within Memorial University of Newfoundland (MUN). The models, featuring third-degree lacerations, were developed collaboratively by MUN MED 3D designer and researcher (CG) and Memorial University faculty members (JS, AG). The models were designed to be sutured during the workshop as a hands-on method to practice what is considered to be a high risk, low occurrence (HALO) procedure, connected to many distressing postpartum complications [8].

This technical report describes the development and validation of an iterative silicone OASIS model based on a previously developed second-degree perineal repair model. This report is part of a larger series of product development and evaluation of silicone perineal repair models. The first technical report, focusing on first and second-degree laceration models, was produced based on findings from the Remote and Rural Conference in St. John’s, Newfoundland in April 2018 [9].

## Technical report

**Figure 1:**
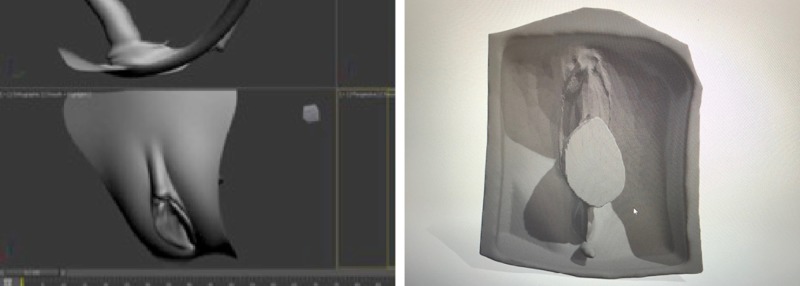
Digital 3D model and inverse mold.

To produce the OASIS silicone models, a pre-existing model from an open-source royalty-free website (https://3dexport.com) was purchased ($10) and converted using Fusion360TM (Autodesk Inc., San Rafael, CA, USA) into a stereolithography (STL) file, which was used to create an inverse mold for the silicone to be poured into (Figure 1) [10].

The creation of 3D printed inverse molds for silicone casting was a technique used by the founding researchers of MUN Med 3D and utilized during the development phase of this project. The 3D rendered mold in the form of a .stl file was transferred using a USB key, to an Ultimaker 3 3D printer (Ultimaker B.V., The Netherlands) and printed using polylactic acid (PLA) filament material. PLA filament is an optimal material for mold making, as it has a rigid texture when printed. The molds were then filled with 250 ml of silicone, including a flesh-toned pigment to give it a realistic appearance.

**Figure 2:**
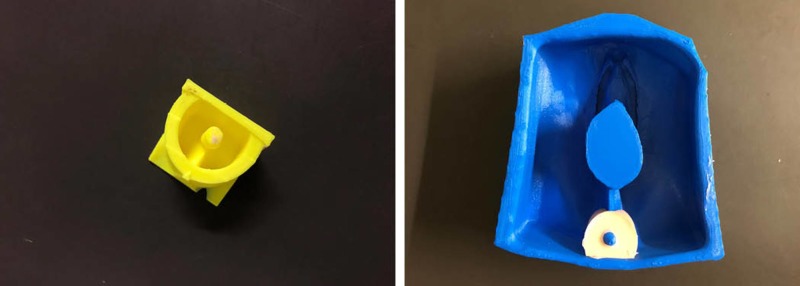
Anal sphincter mold 3D printed and poured separate from the main model.

The anal sphincter was isolated and 3D printed, which was then filled with pigmented silicone. The sphincter was an important feature of the model, as trainees should be able to identify the muscular ring of the anus and be able to reattach the ends prior to the perineal repair (Figure 2).

**Figure 3:**
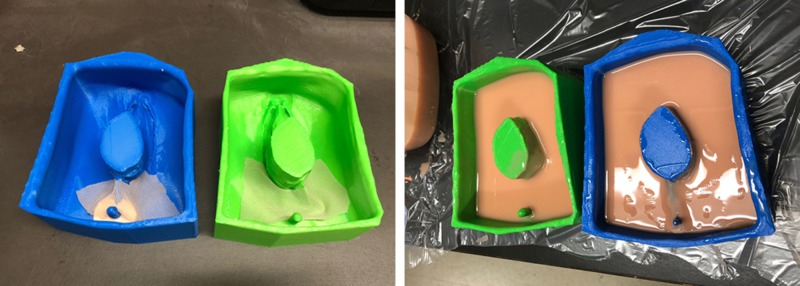
OASIS mold with mesh netting and silicone mixture. OASIS: obstetric anal sphincter injuries

A thin silicone layer was first applied inside the inside bottom of the mold, followed by a small piece of flesh-colored mesh material, fitting the width of the mold. Mesh was added to the perineal plane and around the vaginal canal to ensure the sutures would not tear through the model during OASIS repair. Each silicone mixture required three hours to fully set before being removed from the mold (Figure 3).

Context

**Figure 4:**
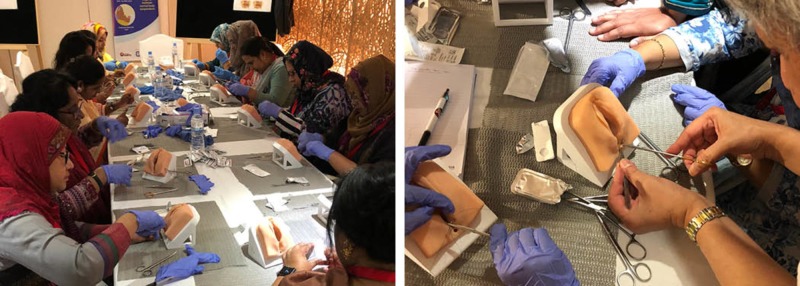
Team Broken Earth’s OASIS repair workshop station. OASIS: obstetric anal sphincter injuries

Team Broken Earth hosted the International Training Course on High-Risk Labour and Delivery Management from September 11 – 13 in Dhaka, Bangladesh, and included 11 Canadian medical educators to host the workshops. The conference attracted a variety of health care students and professionals, specifically family doctors, nurses, obstetrics, and gynecologists, midwives, professors, and students. The three-day course included eight simulation-based training stations designed to advance local knowledge and training in women’s health as related to labor and delivery. The workshops taught delegates how to manage birth complications such as shoulder dystocia, helping babies breathe, the application of uterus compression sutures (for the management of postpartum hemorrhage) and the repair of OASIS. The OASIS repair and uterus compression suturing stations provided opportunities to better understand how to manage third/fourth-degree lacerations and postpartum hemorrhage respectively, which are both linked to a high maternal mortality rate in the country (Figure 4).

The OASIS repair workshop was organized by the Obstetrics and Gynecology (OB/GYN) department at MUN and was facilitated by a medical educator (author AG), an Associate Professor and Chair of Obstetrics at MUN. Following the 45-minute workshops, the participants were provided an option to complete a research feedback survey to assess if the models were effective as a simulation-based training tool for future use.

Input

The equipment provided to trainees included: forceps, suturing scissors, needle drivers, rubber grip placemats, medical grade catgut suture, and the OASIS models. The OASIS models contained the following revisions based on the original study in this series: (a) the addition of a visible anal sphincter muscle of a slightly lighter colour, (b) third/fourth-degree laceration pre-built into the model, (c) flesh-toned colouring, (d) larger vaginal canal indicative of a postpartum condition, (e) mesh built into the silicone at deeper layers to hold the initial interstitial anchor stitch, as well as the perineal plane crown stitch.

Process

On the first day of the conference, a medical facilitator (author AG) led a Powerpoint presentation about perineal OASIS repair. This preceded a session where delegates practiced repair techniques on the OASIS models. During each OASIS repair workshop, the facilitators provided a 10-minute briefing about the specific suturing patterns, as well as a hands-on demonstration of the suturing technique. Throughout the 45-minute session, the facilitators attended to each delegate to answer questions and assist with their techniques.

**Figure 5:**
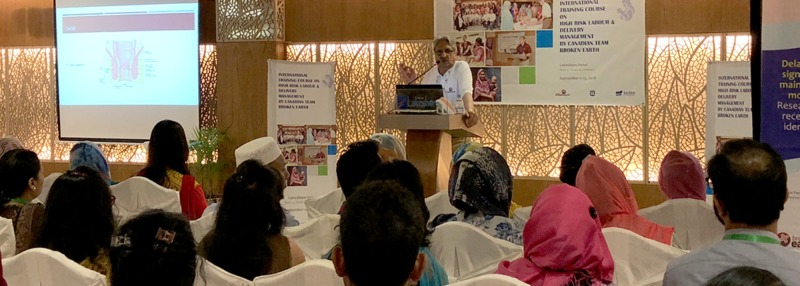
Conference delegates at the International Course for High-Risk Labor and Delivery in Dhaka, Bangladesh September 11-13, 2018.

The models were created from a digital three-dimensional (3D) file (.STL), which was altered and produced using an Ultimaker 3 3D printer to fabricate the molds for the silicone material. The simulation models were tested in eight one-hour workshop sessions with over 120 clinical participants (including OB/GYN clinicians, professors, nurses, midwives, family doctors, and students) held at the Lake Shore Hotel in Dhaka. The same 25 silicone models were used in each workshop, which showed minimal wear after the insertion and removal of the respective amount of sutures. The workshops commenced each day following a 45-minute morning lecture led by a medical facilitator (author AG). Each workshop group consisted of 12-14 participants who provided qualitative feedback about the efficacy of the models as a hands-on training tool for OASIS repair (Figure 5).

Products/outcomes

**Figure 6:**
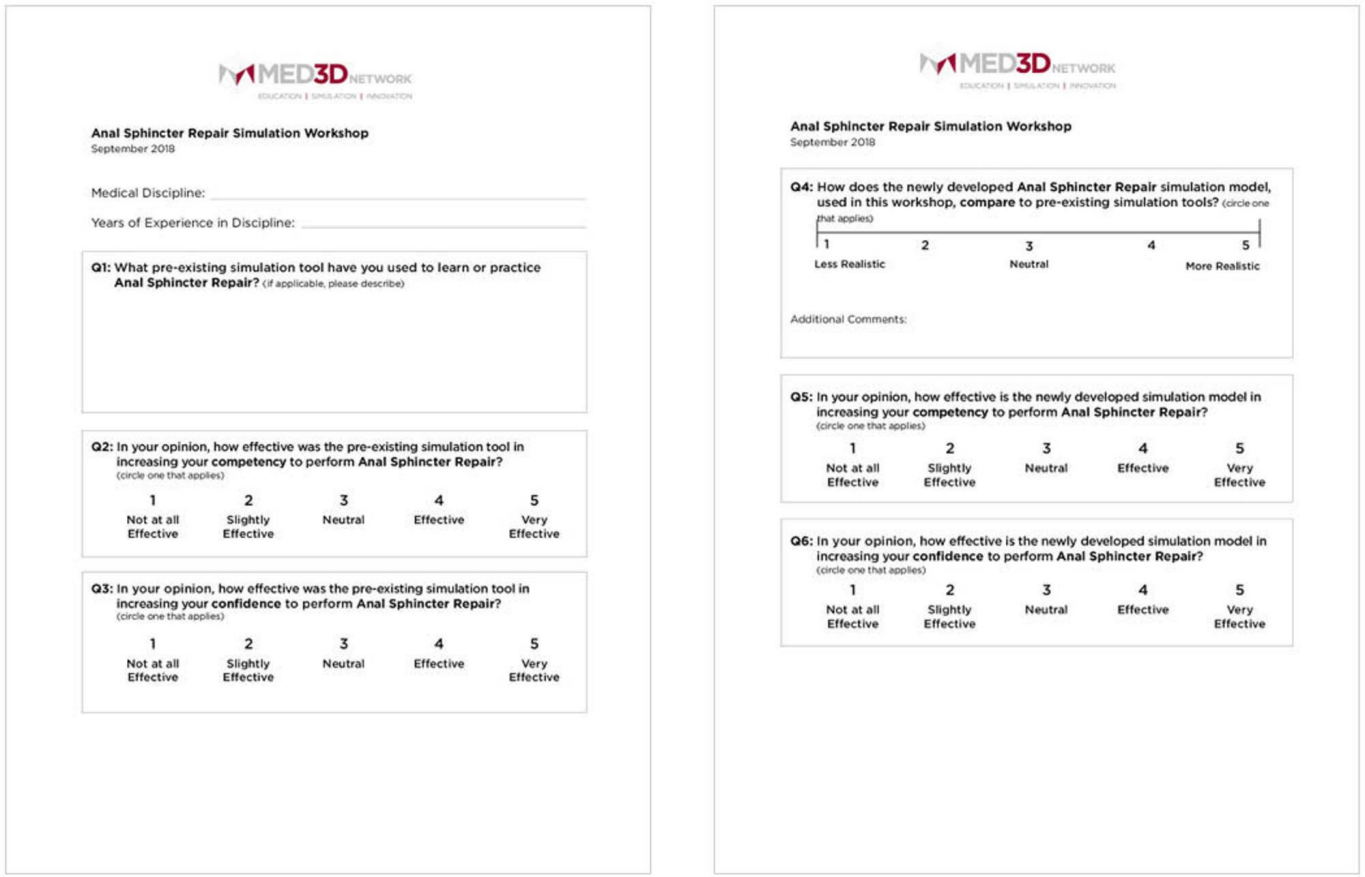
Survey to evaluate the performance of the OASIS models compared to the pre-existing models OASIS: obstetric anal sphincter injuries

**Figure 7:**
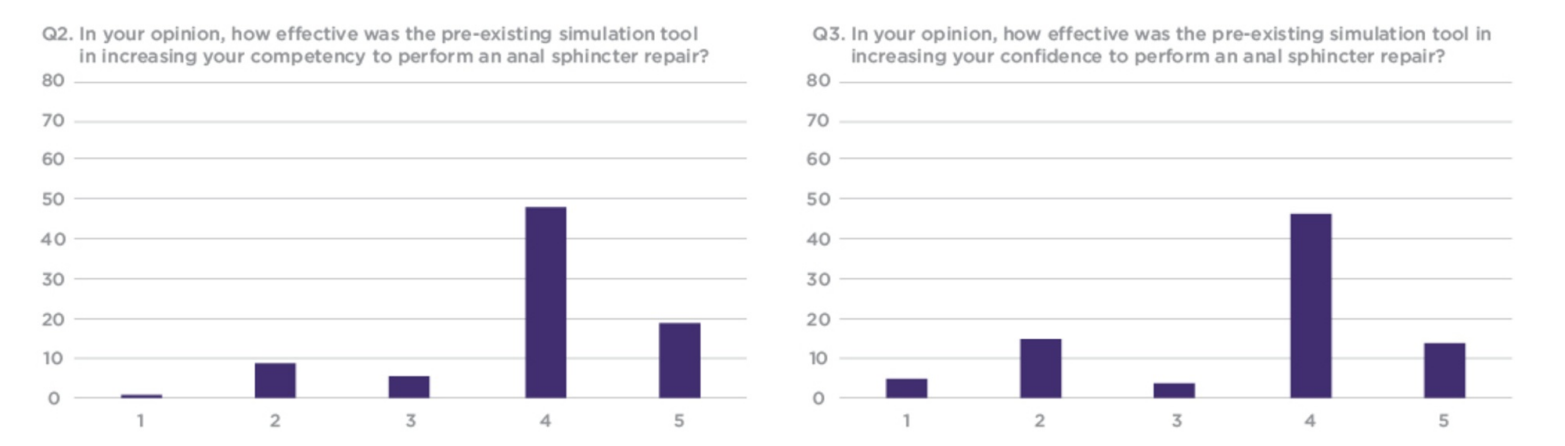
Q2 and Q3 results from workshop participant feedback survey Y Axis: Frequency of responses X Axis: Referring to the response anchors as related to each number respectively in Figure 6

**Figure 8:**
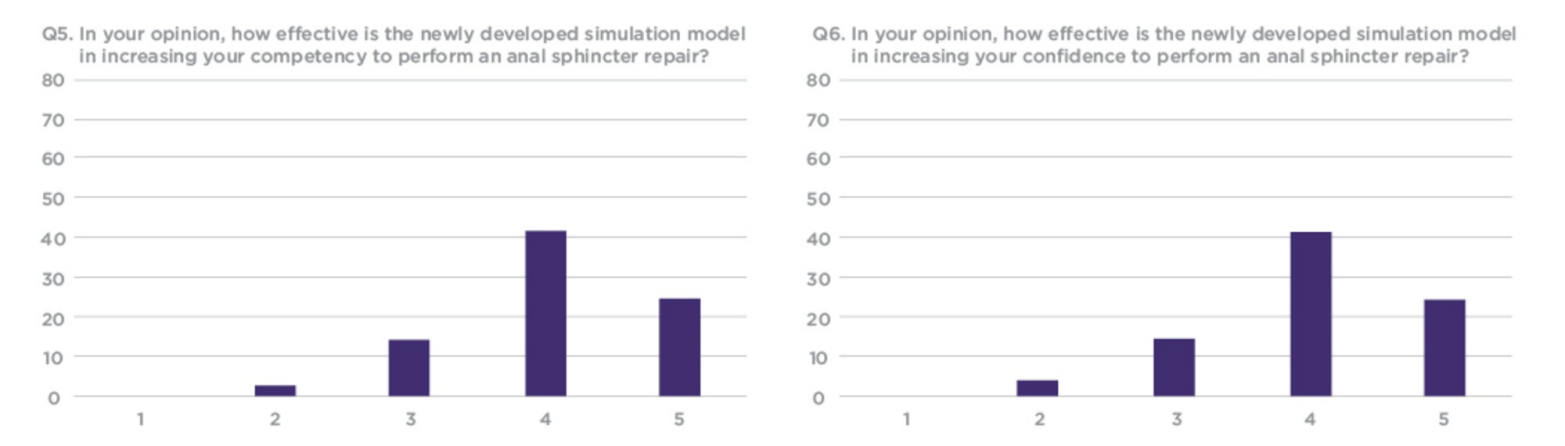
Q5 and Q6 results from workshop participant feedback survey Y Axis: Frequency of responses X Axis: Referring to the response anchors as related to each number respectively in Figure 6

**Figure 9:**
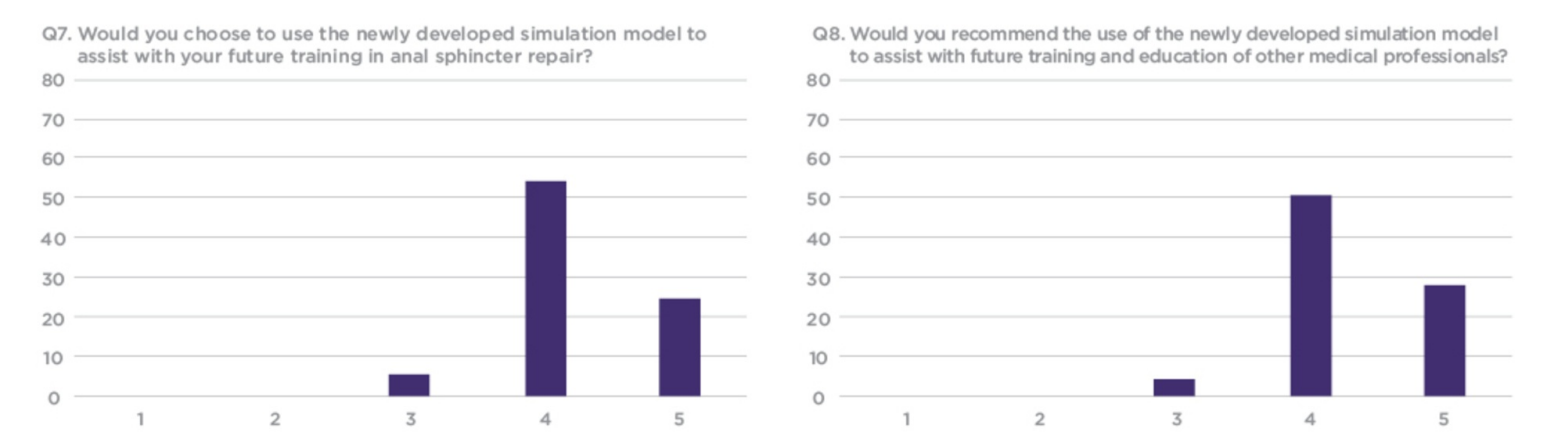
Q7 and Q8 results from workshop participant feedback survey Y Axis: Frequency of responses X Axis: Referring to the response anchors as related to each number respectively in Figure 6

The conference delegates were provided with a research survey to better understand if the OASIS models would be an effective tool for future use in OBS/GYN training programs in Bangladesh. The survey included 10 structured questions - seven questions prompted participants to score 1-5 on a linear Likert scale, one question prompted participants to rate the model on a sliding realism scale, and two open-ended questions prompted feedback regarding the simulation experience. (Figures 6-9).

Out of the 120 workshop participants who evaluated the model, 82 completed the research survey. The results show that a majority of trainees would like to use such models for future training and have not used anything like these previously in their training. The responses indicated that over 50% of participants considered the silicone OASIS models to be an effective tool to increase confidence and competency when performing OASIS repair (Figure 8).

## Discussion

**Figure 10:**
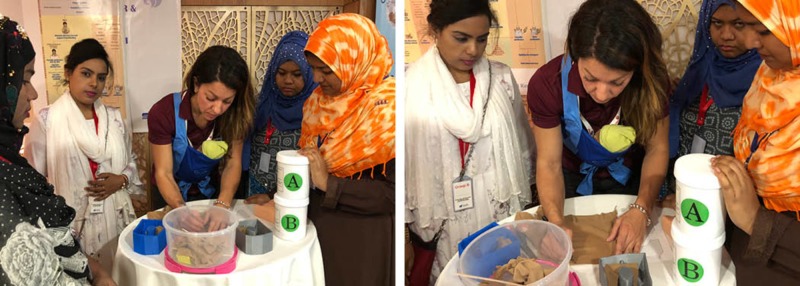
Live silicone mixing and pouring demo during day 3 of the Team Broken Earth training workshop in Dhaka, Bangladesh.

The OASIS silicone model was thought to be superior as compared to traditional didactic learning, by providing an anatomically correct simulation tool to rehearse the repair of the anal sphincter. The models were also very durable in that they showed very little signs of wear after the eight workshop sessions, which rotated over 120 participants. A need was expressed by the workshop participants to have access to such models locally so clinicians and trainees can have ongoing training for OASIS repairs. These were especially important for traditional birthing assistants, who deliver 70% of babies in rural Bangladesh and have very minimal medical training to assist with urgent care deliveries at home [7]. To provide a more accurate context for the simulation, the OASIS models would also have blood and excess tissue surrounding the opening of the vaginal canal during the procedure so trainees can experience an even more advanced simulation scenario. During the conference, silicone was sourced locally in Dhaka so a live mixing demo could be provided during day three of the Team Broken Earth training course (Figure 10).

Currently, the next iteration of the models is underway to advance the aesthetics, in regards to becoming more inclusive with skin tone. The model is also the basis for an innovative fistula injury model under the direction of the faculty of OB/GYN (author AG) at MUN. Mesh will also be added across the back of the revised models so participant’s’ fingers do not puncture the vaginal canal when suturing the models.

**Figure 11:**
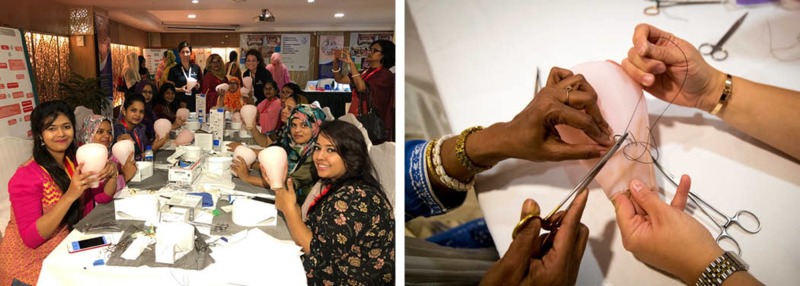
Conference delegates participating in the postpartum B-Lynch uterus compression suturing workshop.

In addition, the silicone uterus workshop models were also produced in a similar fashion as the silicone OASIS models and proved to be an effective tool for simulation training of applying compression sutures (Figure 11). A follow-up technical report will be compiled to analyze the results of such uterus models as well.

## Conclusions

Silicone OASIS models, created from a 3D printed mold, are an effective method for training midwives, obstetric and gynecology clinicians, as well as rural, at-home birthing attendants in developing countries. The molds, which can be 3D printed locally in Bangladesh at the University of Dhaka, where a 3D printer is currently located, are a cost-effective means to produce repeatable molds and resulting models. Silicone is considered a local raw material in Bangladesh (http://www.sc.com.bd/silicone-rubber-female-body-organs-casting-medical-grade-liquid-silicone/) and can be purchased for approximately 1,480 taka (which is the equivalent of approximately $22 CAD).

The results of the workshops with 180 participants during the Team Broken Earth International Training Workshop on High-Risk Labour and Delivery Management were very positive with regards to the effective nature of the silicone models. The workshops included students, professors, biomedical engineers, doctors, nurses, and midwives, who completed the research survey to determine the efficacy of such models for ongoing training purposes. The results revealed the need for such workshops and suturing models, especially for rural birthing attendants, who often assist at-home births, without medical training. An additional consideration for rural developing communities, as observed (by author CG), is to develop OASIS-specific sutures that are affordable and accessible to rural families. With rural at-home births connected to such a significant maternal mortality rate, it would be ideal to provide such developing countries with a low-cost solution to repair OASIS at home as a way to lower the instances of long-term health complications and fatalities connected to improper care during delivery management. More research is required to determine how to design and produce OASIS-specific sutures and silicone models at the most cost-effective price point possible so ongoing training can occur globally.

## References

[1] National Academy of Sciences (2009). The US Commitment to Global Health: Recommendations for the Public and Private Sectors. https://www.ncbi.nlm.nih.gov/books/NBK23788/.

[2] Philibert I (2009). International medical education outreach: benefits for US medical education and practice. J Grad Med Educ.

[3] Temmerman M, Khosla R, Laski L, Mathews Z, Say L (2015). Women’s health priorities and interventions. BMJ.

[4] (2018). Dr. Andrew Furey. Team Broken Earth. http://andrewfurey.ca/team-broken-earth.

[5] (2018). Team Broken Earth. https://brokenearth.ca/about/.

[6] Sarker BK, Rahman M, Rahman T, Hossain J, Reichenbach L, Mitra DK (2018). Reasons for preference of home delivery with traditional birth attendants (TBAs) in rural Bangladesh: a qualitative exploration. Plos One.

[7] Fronczak N, Arifeen SE, Moran AC, Caulfield LE, Baqui AH (2007). Delivery practices of traditional birth attendants in Dhaka slums, Bangladesh. J Health Popul Nutr.

[8] Harvey MA, Pierce M (2015). Obstetrical anal sphincter injuries (OASIS): prevention, recognition, and repair. J Obstet Gynaecol Can.

[9] Goudie C, Shanahan J, Gill A (2018). Investigating the efficacy of anatomical silicone models developed from a 3D printed mold for perineal repair suturing simulation. Cureus.

[10] (2018). Female genital realistic vagina, 3D model. https://3dexport.com/3dmodel-female-genital-realistic-vagina-79891.htm.

